# Tuberculous Spondylodiscitis in South East Tunisia: Features and Predictive Factors of Poor Prognosis

**DOI:** 10.7759/cureus.93635

**Published:** 2025-10-01

**Authors:** Hamida Kwas, Sabrine Mejdoub Fehri, Moez Ben Ayed, Oumayma Aggoumi, Harish Rangareddy, Hayfa Rajhi

**Affiliations:** 1 Pulmonology Department, University of Sfax, Faculty of Medicine of Sfax, Gabes University Hospital, Gabes, TUN; 2 Pulmonology Department, Faculty of Medicine of Sfax, Gabes University Hospital, Gabes, TUN; 3 Orthopedics Department, University of Sfax, Faculty of Medicine of Sfax, Gabes University Hospital, Gabes, TUN; 4 Biochemistry Department, Haveri Institute of Medical Sciences, Haveri, IND; 5 Analysis Laboratory Research, Gabes University Hospital, Gabes, TUN

**Keywords:** diagnosis, prognosis, treatment, tuberculosis, tuberculous spondylitis

## Abstract

Background: Tuberculous spondylodiscitis is a severe form of tuberculosis (TB) infection. The study aims to determine clinical and radiological features and treatment outcomes of tuberculous spondylodiscitis in southeast Tunisia and to identify factors associated with poor prognosis.

Methods: A retrospective, single-center study was conducted over a period of 16 years (2008-2023). All patients treated for tuberculous spondylodiscitis were included. Data were collected on demographic, clinical, and radiological features, as well as treatment regimens and outcomes. Multivariable logistic regression analysis was performed to identify factors independently associated with unfavorable outcomes.

Results: Twenty-six patients were included in our study, with an average age of 46 years ± 15.69. A female predominance was observed (18 women and eight men). The average symptom duration was 5.6 months ± 4.35. Spinal pain was the revealing functional sign (80.7%). The diagnosis of TB was confirmed in 65.% of patients. Anti-TB treatment was prescribed in all patients with a mean duration of 13.88 ± 3.789 months (10-18 months). At one-month follow-up, 69.2% of patients had a favorable outcome. Independent predictors of poor prognosis included age ≥ 65 years (odds ratio (OR): 2.26), diagnostic delay ≥ 5 months (OR: 1.33), presence of spinal cord compression (OR: 13.9), and initial C-reactive protein (CRP) level ≥ 50 mg/L (OR: 1.24).

Conclusion: In patients with tuberculous spondylodiscitis, the outcome at one month of follow-up was favorable in only 69.2%. Advanced age, delayed diagnosis (≥5 months), spinal cord compression, and elevated CRP levels (≥50 mg/L) emerged as independent predictors of poor outcomes at one-month follow-up. Further studies are required, particularly in TB-endemic countries, to investigate other factors associated with unfavorable outcomes of tuberculous spondylodiscitis.

## Introduction

Tuberculosis (TB) is an ancient disease that is currently re-emerging as a public health problem worldwide. In 2022, TB was the second leading cause of death from a single infectious agent, following the COVID-19 pandemic, and caused almost twice as many deaths as HIV/AIDS [1]. Although TB frequently affects the lungs, extrapulmonary forms are increasingly observed. Osteoarticular TB accounts for 10% of extra-pulmonary TB and 3-5% of all forms of TB combined [2]. The exact prevalence of tuberculous spondylodiscitis in most countries is not known. In countries with a higher incidence of TB, the prevalence is expected to be proportionately high. Tuberculous spondylodiscitis occurs in approximately 1-2% of people with TB [3,4]. Tunisia has an intermediate endemicity for TB with a reported incidence of 38 per 100,000 inhabitants in 2023. Lung involvement represents 38% of all forms of the disease in 2017, whereas extrapulmonary involvement represents 62% of cases. The incidence of tuberculous spondylodiscitis seems to be high. This is considered the most severe form due to its proximity to neural structures [5,6]. Up to the present day, tuberculous spondylodiscitis continues to arouse interest due to its severe complications, namely, spinal deformities, neurological deficits, and paravertebral abscesses, which may result in lasting sequelae and relapse. Despite the frequency and severity of tuberculous spondylodiscitis, data regarding its management, particularly in identifying patients at high risk of poor outcomes, are scarce. In this study, we sought to characterize the clinical, biological, and radiological features, as well as outcomes after treatment, and to identify factors associated with unfavorable outcomes of tuberculous spondylodiscitis in southeast Tunisia, in which the disease is endemic.

## Materials and methods

Study design and type of study: A retrospective, single-center study was conducted over a 16-year period (January 2008 to December 2023) in the University Hospital of Gabes, southeast Tunisia. This study was carried out in the pulmonology and orthopedics departments. Data were collected on demographic, clinical, and radiological features, as well as treatment regimens and outcomes.

Study population: We included in the present study patients who were treated for tuberculous spondylodiscitis. Inclusion criteria are as follows: age greater than or equal to 14 years, living in the city of Gabes, and being treated in Gabes University Hospital. Patients not residing in Gabes and those not treated at the Gabes University Hospital are excluded from the study. The diagnosis of TB was retained in the presence of bacteriological evidence following isolation of *Mycobacterium tuberculosis *(*M. tuberculosis*) and/or histological evidence following the identification of a granuloma with caseous necrosis on examination of the disco-vertebral biopsy or the biopsy of an abscess. In some cases, without bacteriological or histological proof, the diagnosis of tuberculosis is made in the presence of clinical and radiological manifestations and response to anti-TB treatment. Patients were excluded from the study if they were non-compliant with their treatment, if they were lost to follow-up, or if they died from other causes during the anti-TB treatment period.

Data collection: Data including age, sex, smoking, bacille Calmette-Guerin (BCG) vaccination at birth, if contact with a person with TB infection, personal history of TB, sites of TB infection, functional signs, the consultation time (the time between the onset of symptoms and the day of consultation), laboratory findings, findings from standard X-rays, chest X-ray (CXR), spinal computed tomography scan (CT scan) and magnetic resonance imaging (MRI), biopsy findings, results of smears and cultures, medical treatment, types and duration of treatment, surgical treatment, and outcomes were collected from the case notes of patients. Patients were assessed on day (D) 7, D15, D21, D30, at three months, and at the end of the anti-TB treatment, according to the Tunisian guide recommendations for the management of infectious spondylodiscitis [6]. Outcome at one month of treatment was considered good in the presence of the following criteria: apyrexia, weight gain, recovery of appetite, disappearance of spinal pain, decrease in C-reactive protein (CRP) level, regression of collection images, and the absence of vertebral deformities, neurological impairment, or sepsis. Outcome was considered unfavorable in the absence of one of these clinical, radiological, and biological criteria and/or in the presence of neurological sequelae or spinal bone deformities.

Ethical considerations: This study was approved by the Ethics Committee of Gabes University Hospital.

Statistical analysis: Statistical analyses were performed using Statistical Product and Service Solutions (SPSS, version 26; IBM SPSS Statistics for Windows, Armonk, NY). A p-value of <0.05 was considered statistically significant. Continuous variables were presented as means with standard deviations (SD), while categorical variables were summarized as absolute and relative frequencies (percentages). Multivariate logistic regression analysis was conducted to identify factors associated with unfavorable outcomes, with variables showing p < 0.1 in univariate analysis included in the model.

## Results

**Figure 1:**
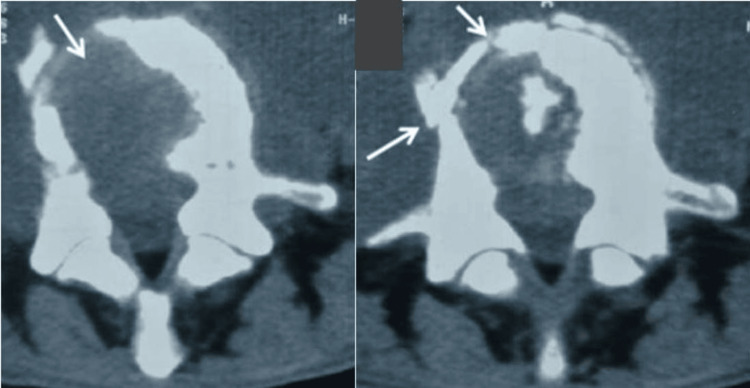
Lumbar spine CT scans: osteolysis of the vertebral body of L5 with bone fragmentation and anterior and posterior cortical rupture

In our study, 26 cases of tuberculous spondylodiscitis were recorded during the period spanning from January 2008 to December 2023, representing 2% of reported cases of TB. During the study period, the average annual incidence of spinal TB was two cases per year. The average age of our patients was 46 years ± 15.69 (ranging from 24 to 84 years), with a predominance of females (69.2%). Twelve patients (46.1%) had a history of smoking, and 18 patients (69.2%) consumed raw milk. Most patients (69.2%) received Bacillus Calmette-Guérin (BCG) vaccination at birth. There were underlying diseases in 18 patients: diabetes (eight), high blood pressure (six), connective tissue disease with steroid treatment (two), chronic obstructive pulmonary disease (four), and asthma (two). Four patients had a history of lymph node TB. The average consultation time was 5.6 months ± 4.35 (12 days and 17 months). Clinical symptoms varied widely. However, spinal pain was present in the majority of our patients (80.7%). Signs of TB impregnation, such as fever, weight loss, anorexia, and asthenia, were present in 69.2% of patients. Spinal stiffness was noted in 38.4% of patients. Neurological deficit was noted in 14 patients (53.8%), with paraparesis in eight patients, divided according to Frankel's grading into grade A (four cases), grade B (three cases), and grade D (one case). Six patients had paraplegia related to spinal cord compression. Standard X-ray of the spine, performed in all patients as a first-line procedure, showed abnormalities suggestive of spondylodiscitis in 53.8% of cases: erosion of the vertebral endplates (34.6%), paravertebral spindle appearance (19.2%), disc pinch (19.2%), and vertebral fracture (7.6%). A spinal CT scan was performed in 18 patients (69.2%), showing paravertebral abscess (26.%), epiduritis (38.4%), osteolysis of the vertebral body (30.7%) (Figure 1), disc pinch (19.2%), mirror geode (15.3%), and erosion of the vertebral endplates (19.2%). MRI of the spinal cord was requested in most patients (84.6%). It was abnormal in all cases (Table 1).

**Table 1:** Radiologic manifestations of tuberculous spondylodiscitis discovered on MRI Categorical variables were summarized as absolute and relative frequencies.

MRI abnormalities	Number of patients	Percentage (%)
Epiduritis	21	80.7
Paravertebral abscess	10	38.4
Disc pinch	5	19.2
Diffuse or circumferential signal increase in sp T1 Gado	6	23
Thickness of pre-spinal soft tissues	8	30.7
Spinal cord compression	6	23
Osteolysis of the vertebral body	10	38.4

**Figure 2:**
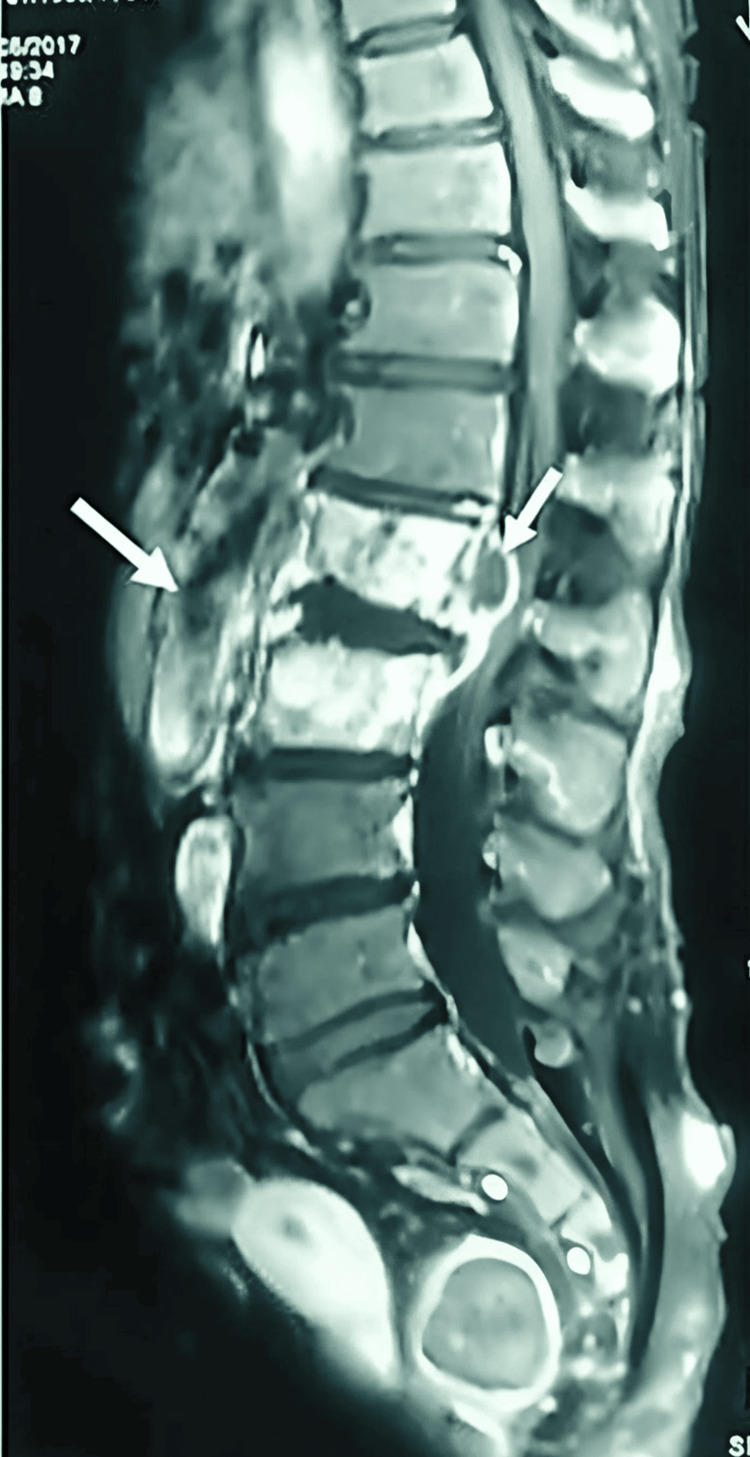
Sagittal view of a T2-weighted magnetic resonance imaging showing Short-Tau Inversion-Recovery (STIR) hyperintensity of the L2 and L3 vertebral bodies with highly enhancing STIR hyperintensity of the L1-L2 disc, raising the suspicion of contiguous discitis associated with anterior epidural abscesses extending over 4 cm

**Figure 3:**
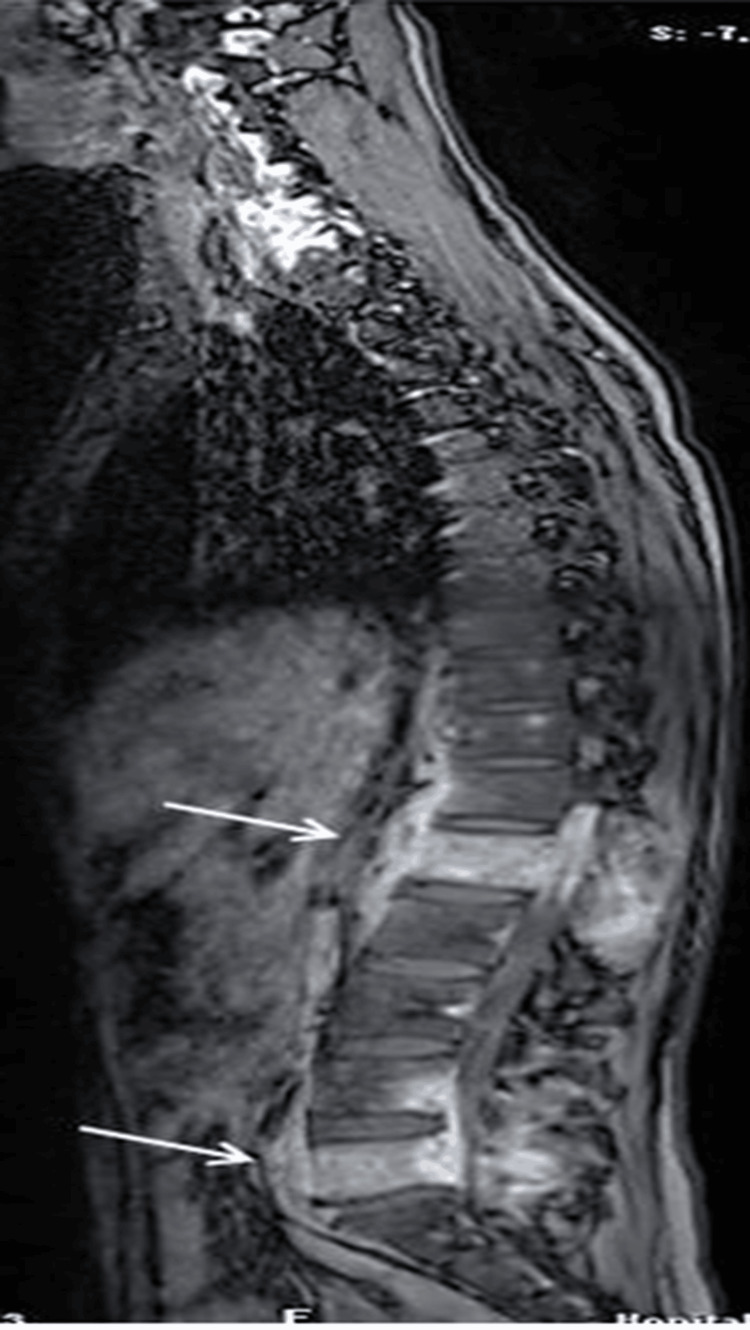
Sagittal view of magnetic resonance imaging showing inflammatory involvement of the vertebral body of the L1 and the posterior arches with large left paravertebral abscesses At L5 level: paravertebral abscesses with compression of the cauda equina nerve roots.

Chest X-ray (CXR) performed systematically in all patients showed abnormalities in two cases. The distribution of involved vertebrae showed a predilection for the lower thoracic and lumbar vertebrae (Figures 2-3).

**Figure 4:**
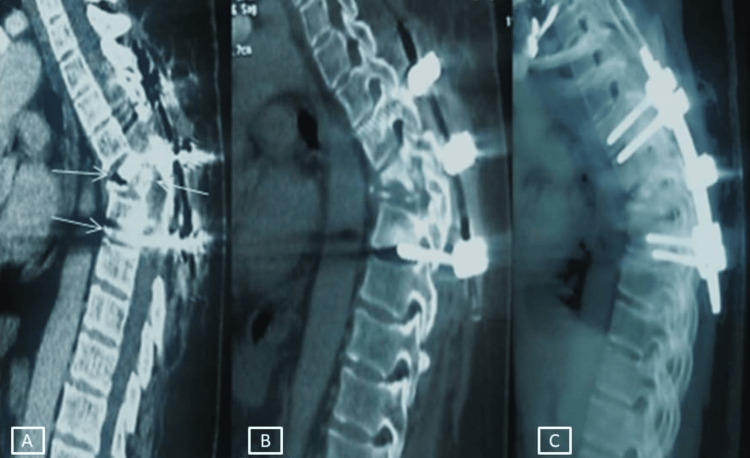
Computed tomography of the dorsal (thoracic) spine showing sequelae of spondylodiscitis: D5-D6 dorsal kyphosis Post-surgical changes following decompressive spinal laminectomy, demonstrating worsening of thoracic kyphosis with osteosynthesis.

Eighteen patients (69.2%) had a lumbar focus, and eight (30.8%) had a thoracic focus. A tuberculin skin test was performed in 11 patients (42.3%) and was positive in four cases. Admission blood tests showed a biological inflammatory syndrome in 65.3% of cases, with an increase in CRP level, with a mean concentration of 87.2 ± 17.6 mg/L (12-133 mg/L), and white blood cell count (mean: 14.210 ± 4.625 cell/mm^3^). Normochromatic normocytic anemia was found in 12 patients (46.1%). Sputum tests for Koch’s bacillus were requested in all patients and were negative in all. *M. tuberculosis* testing was also carried out on urine in six patients and was negative in all cases. Of the different samples taken (biopsy puncture of paravertebral abscess in seven patients, disco-vertebral biopsy CT-guided in eight patients, and surgical biopsy made after decompression laminectomy in four patients), the diagnosis of TB was confirmed after isolation of *M. tuberculosis *by culture and/or a PCR gene amplification test in 10 patients (38.4%). All positive cultures were sensitive to first-line TB drugs. The diagnosis was confirmed by histopathological evidence in seven patients (26.9%), showing tuberculoid granuloma with caseous necrosis. For the remaining patients, the diagnosis of TB was made on the basis of clinical, radiological, and evolutionary evidence. For the two patients with associated pulmonary TB, only one sputum sample revealed acid-fast bacilli (AFB) on microscopy. The diagnosis time varied from 25 days to five months, with an average of 3.8 months. As factors favoring the development of TB infection, we noted a poor nutritional status in six patients, diabetes in eight patients, anemia in 12 patients, long-term treatment with corticosteroids in two patients, a history of lymph node TB in four patients, and smoking in 12 patients. Routine HIV testing was performed on all patients and was found to be negative in all cases. All patients were put under anti-TB treatment (first-line anti-TB chemotherapy based on isoniazid (INH), rifampicin (RIF), pyrazinamide (PZA), and ethambutol (EMB)), all in the form of fixed-dose combinations. All patients received quadruple treatment for two months, followed by maintenance treatment with INH and RIF for a total duration of between 10 and 18 months, with a mean of 13.88 ± 3.789 months. The extension of treatment is influenced by the clinical and radiological course. In addition to antibacillary treatment, corticosteroid therapy was prescribed for patients with neurological deficits (53.8% of patients). Medications used are dexamethasone during hospitalization (1 mg/kg/day) and then prednisolone (0.5 mg/kg/day) for one month with a very gradual reduction. All patients had spinal immobilization for three months until radiological consolidation of the pottic focus. Surgical treatment has been indicated in 11 patients (42.3%). In these patients, a decompressive spinal laminectomy with osteosynthesis was necessary in four patients because of the presence of neurological deficits on clinical examination and an epidural abscess causing spinal cord compression on MRI (Table 1). Surgical drainage of a paravertebral abscess was performed in seven patients (26.9%). Rehabilitation was an essential therapeutic adjunct for all patients with deficits. In the patients operated on, we recorded a spectacular recovery of the neurological deficit in all cases. It should be noted that four of these patients showed worsening of their kyphosis after laminectomy (Figure 4).

We noted a partial regression of the inflammatory phenomena with the persistence of paravertebral collection in two patients. Thus, the anti-TB treatment was prolonged for up to 22 months in both cases and stopped when the lesions remained stable. None of our patients had complications related to anti-TB treatment. At one month of follow-up, only 69.2% of patients had a favorable clinical outcome, and 61.5% had a CRP decrease. We recorded decubitus-related complications such as thrombophlebitis in two patients and sacral pressure sores in two others. One patient died due to bilateral pulmonary embolism. Among the clinical, biological, and radiological parameters, only age ≥ 65 years (p = 0.036), diagnostic delay ≥ 5 months (p < 0.001), spinal cord compression (p = 0.005), and initial CRP level ≥ 50 mg/l (p = 0.026) were found as predictive factors of poor outcome at one month of follow-up (Table 2).

**Table 2:** Prognostic factors for tuberculous spondylodiscitis The significance of correlations between datasets was determined using Pearson’s ‘r’ values.

Prognostic Factor	p-value	Odds ratio ((95% Confidence interval)
Age ≥ 65 years	0.036	2.26 (1.055-4.716)
Diagnostic delay ≥ 5 months	<0.001	1.33 (1.11-1.88)
Spinal cord compression	0.005	13.9 (2.2-81.9)
Initial CRP level ≥ 50 mg/L	0.026	1.24 (1.12-1.45)

## Discussion

TB remains endemic in all Tunisian regions, particularly in southeast Tunisia. Pulmonary TB is the most common form of TB, but extra-pulmonary forms are increasingly common. Tuberculous spondylodiscitis is the most serious form of extra-pulmonary TB. In the present study, we first reported the clinical, biological, and radiographic manifestations, as well as treatment outcomes of tuberculous spondylodiscitis, and then studied the factors associated with poor early outcomes. Thus, we showed that old age, diagnostic delay ≥ 5 months, spinal cord compression, and initial CRP level ≥ 50 mg/L are predictive factors of poor outcome in TB spondylodiscitis.

In our study, adults and older patients were affected, and the average age was 46 years. This is consistent with the results of other Tunisian studies [7,8] and with the results of studies carried out in developed countries [9-11]. We found in our study that advanced age is correlated with poor outcomes (p = 0.036). The same findings have been reported by a Tunisian study [8]. In our study, there was a predominance of women. These results are consistent with those of most studies [10-12]. The existence of a history of TB reported in different studies varies from 4.6% to 28.2% [13-15]. Four of our patients had a history of lymph node TB. The time to diagnosis of TSPD is generally long, ranging from 3 to 12 months [16,17]. In our study, diagnosis time varied from 25 days to five months, with an average of 3.8 months. We reported in our study that diagnostic delay ≥ 5 months was associated with disease severity (p < 0.001), which has not yet been reported in the literature, requiring further studies with a larger sample size of patients to better elucidate this association. The clinical presentation of tuberculous spondylodiscitis is highly variable. However, spinal pain remains the most frequent reason for consultation in the various studies [18], which is consistent with our results. Pain may be mechanical, inflammatory, or mixed. In the present study, a biological inflammatory syndrome was noted in 65.3% of cases with increased CRP level and white cell count, which is consistent with the results of several studies. We found that the initial CRP level ≥ 50 mg/L was correlated with a poor outcome (p = 0.026), but this has not been reported in the literature. In our study, normochromic normocytic anemia was present in 46.1% of patients. Anemia is known as a risk factor for infectious diseases, particularly TB [19]. However, we did not find an association between anemia and an unfavorable outcome of tuberculous spondylodiscitis. Although standard X-rays should be used as a first line, they do not provide an early diagnosis of spondylodiscitis, as lesions will only be visible once more than 30% of the vertebra has been destroyed [2]. A CT scan shows abnormalities earlier than standard X-rays. Its sensitivity reaches 100% [2,20]. Owing to advances in imaging, MRI has revolutionized the diagnosis of infectious spondylodiscitis by being a non-invasive examination and providing rapid diagnosis [2]. In our study, several types of radiological abnormalities were observed, such as epiduritis, paravertebral abscess, spinal cord compression, and osteolysis of the vertebral body. Furthermore, we found that the presence of radiological evidence of spinal cord compression was correlated with an unfavorable outcome (p = 0.005). It has been shown that greater destruction of the spine and a significant number of affected vertebrae are predictors of an unfavorable outcome [21,22]. Culture of samples from either abscess puncture or disc-vertebral biopsy is the most sensitive method for isolating the strain of *M. tuberculosis *in order to establish the bacteriological diagnosis of tuberculous spondylodiscitis. However, with the development of new diagnostic techniques based on molecular biology, the microbiological diagnosis of spondylodiscitis has become easier and faster. In our study, microbiological diagnosis of tuberculous spondylodiscitis was obtained in 10 patients (38.4%). In practice, it is difficult to make a definitive diagnosis of tuberculous spondylodiscitis in most cases, and the diagnosis is usually based on radioclinical evidence, which often leads to a preference for therapeutic tests. The therapeutic management of tuberculous spondylodiscitis remains controversial between the different schools of thought. However, anti-TB treatment combined with surgery is the most commonly used approach. Medical treatment of tuberculous spondylodiscitis should be started early in the presence of obvious clinical and radiological signs and even before the bacteriological diagnosis has been established [3]. Indications for surgery are currently decreasing, given the effectiveness of medical treatment and the significant number of complications associated with surgery. Surgical treatment is indicated in patients with refractory disease, severe kyphosis, neurological deficits, when the spine is unstable, and in cases of large abscesses that are resistant to medical treatment [11,23]. In Africa, surgical treatment is customary, given the extent of the lesions at the diagnostic stage. This is the same approach adopted by surgeons in the Far East, who frequently operate for tuberculous spondylodiscitis. Corticosteroid therapy is another therapeutic component with significant contributions to anti-TB treatment. It is primarily prescribed in cases of epiduritis and spinal cord compression leading to Pott's paraplegia. Corticosteroids help reduce mortality, sequelae, and complications; expedite healing; and improve prognosis.

Despite its limitations (retrospective and conducted at a single center with a small sample size), our study allowed us to identify new predictive factors for poor outcome of tuberculous spondylodiscitis.

## Conclusions

Tuberculous spondylodiscitis is a rare but severe form of TB infection, which may lead to severe deformity and early or late neurological complications. The management of this disease is difficult in some cases. In this study, the outcome at one month of follow-up was favorable in only 69.2%. Advanced age, delayed diagnosis (≥5 months), spinal cord compression, and elevated CRP levels (≥50 mg/L) emerged as independent predictors of poor outcomes at one-month follow-up. Further studies are required, particularly in TB-endemic countries, to investigate other factors associated with unfavorable outcomes of tuberculous spondylodiscitis.
